# Editorial: The pathogenesis and intervention of sleep disorders

**DOI:** 10.3389/fnins.2023.1176932

**Published:** 2023-04-27

**Authors:** Jianwei Shuai

**Affiliations:** Wenzhou Institute and Wenzhou Key Laboratory of Biophysics, University of Chinese Academy of Sciences, Wenzhou, China

**Keywords:** sleep disorders, chronic insomnia, obstructive sleep apnea, traumatic brain injury, opioid

Sleep disorders are a growing concern that affects a significant portion of the population and can lead to severe health consequences, including depression, anxiety, and cardiovascular disease. While notable advancements have been made in the diagnosis and treatment of sleep disorders, challenges persist in understanding their underlying mechanisms, and identifying effective interventions. The pathogenesis of sleep disorders is complex and multifactorial, involving both genetic and environmental factors. Therefore, understanding the mechanisms of sleep disorders and identifying effective interventions is crucial for improving diagnosis and treatment. This Research Topic includes five papers that provide insight into current research on the pathogenesis and intervention of sleep disorders.

Dai et al. conducted a study to investigate the effects of electrostatic therapy on nighttime sleep and daytime symptoms in patients with chronic insomnia. The therapy was found to be effective in improving both sleep quality and daytime symptoms, with significant improvements seen in sleep duration, sleep latency, and sleep efficiency. Additionally, patients reported feeling less fatigued and more alert during the day. While the study was limited by its small sample size and lack of a control group, it provides promising evidence for the use of electrostatic therapy as a non-invasive and non-pharmacological treatment option for chronic insomnia.

In another study, Shi et al. used electroencephalography (EEG) to investigate the neural activity in patients with chronic insomnia during nighttime sleep and multiple sleep latency tests. The results showed that these patients had elevated beta activity during both recordings, indicating increased cortical activation and hyperarousal. Additionally, the study revealed a positive correlation between beta activity during nighttime sleep and subjective ratings of insomnia severity. These findings suggest that chronic insomnia may be associated with a state of hyperarousal that persists throughout the day and night, contributing to sleep difficulties. The study highlights the importance of further research into the underlying neurophysiological mechanisms of chronic insomnia.

Obstructive sleep apnea (OSA) is a common sleep disorder that is associated with a higher risk of cognitive impairment. In a study by Shu et al., the integrity of cerebellar-prefrontal cortical pathways was examined in obstructive sleep apnea (OSA) patients with and without mild cognitive impairment (MCI) (Shu et al.). Using diffusion tensor imaging (DTI) and tractography, the study revealed that both OSA groups had significantly reduced fractional anisotropy and increased mean diffusivity in the cerebellar-prefrontal cortical pathways compared to healthy controls. However, the OSA patients with MCI had even greater abnormalities in these pathways than those without MCI. These findings suggest that disrupted cerebellar-prefrontal cortical pathways may contribute to cognitive impairment in OSA patients, highlighting the importance of early detection and treatment of OSA to prevent further cognitive decline.

Traumatic brain injury (TBI) is a common cause of sleep disorders, and the brain-gut axis has been implicated in the regulation of sleep-wake cycles. Zhanfeng et al. investigated the role of intestinal flora in regulating sleep disorders in patients with TBI. The study found that the composition of intestinal flora was significantly altered in TBI patients with sleep disorders compared to healthy controls. Moreover, treatment with probiotics improved sleep quality and reduced daytime sleepiness in TBI patients. This study suggests that targeting the gut microbiota may be a promising approach for managing sleep disorders in patients with TBI.

While Opioids are commonly used for pain management, they can also disrupt sleep architecture and lead to sleep disorders. The article by Bergum et al. provides a comprehensive overview of the impact of opioid use on sleep, including its effects on sleep architecture, respiratory function, and the development of sleep-related breathing disorders. The authors also discuss the potential mechanisms underlying these effects, such as changes in neurotransmitter systems and respiratory drive. The article highlights the need for clinicians to be aware of the potential for opioid-induced sleep disorders and to monitor patients for sleep-related breathing issues. The authors suggest that more research is needed to better understand the complex interactions between opioids, sleep, and breathing, and to develop more effective strategies for managing opioid-induced sleep disorders.

In conclusion, sleep disorders pose a major public health concern, necessitating the implementation of effective prevention and treatment interventions. Recent research has explored the pathogenesis and intervention of sleep disorders, revealing important insights into their underlying mechanisms. These researches include the examination of neural dynamics, brain network characteristics, genetics, and the gut microbiome, which have illuminated the complex interactions that contribute to the development of sleep disorders. Innovative interventions, such as electrostatic therapy and targeting retinal mechanisms, have shown potential as novel treatment avenues for the treatment of sleep disorders.

Personalized sleep medicine is a future direction that may become a reality with further research and development, enhancing the diagnosis and treatment of sleep disorders and related diseases. To achieve this, future research should validate these findings in larger cohorts, using a multidisciplinary approach to address the complex interactions between neural, genetic, and environmental factors that contribute to sleep disorders. This approach will allow the development of personalized interventions that consider each individual's unique sleep profile, thereby improving the overall effectiveness of sleep medicine. Additionally, the development of digital health tools that can monitor and manage sleep quality and quantity may offer a convenient and accessible solution for individuals with sleep disorders.

Finally, sleep disorders represent a significant public health challenge, and it is crucial to continue advancing our understanding of their underlying mechanisms. The findings from recent research offer promising insights into potential targets for developing personalized interventions, and future studies should focus on further validating these findings and incorporating a multidisciplinary approach to improve the overall efficacy of sleep medicine.

## Author contributions

JS wrote the paper.

